# Correlation of diffusion tensor tractography with obstructive sleep apnea severity

**DOI:** 10.1002/brb3.3541

**Published:** 2024-05-21

**Authors:** Bong Soo Park, Dong Ah Lee, Ho‐Joon Lee, Jinseung Kim, Junghae Ko, Won Hee Lee, Jiyae Yi, Kang Min Park

**Affiliations:** ^1^ Departments of Internal Medicine, Haeundae Paik Hospital Inje University College of Medicine Busan South Korea; ^2^ Departments of Neurology, Haeundae Paik Hospital Inje University College of Medicine Busan South Korea; ^3^ Departments of Radiology, Haeundae Paik Hospital Inje University College of Medicine Busan South Korea; ^4^ Department of Family Medicine, Busan Paik Hospital Inje University College of Medicine Busan Republic of Korea; ^5^ Department of Neurosurgey, Busan Paik Hospital Inje University College of Medicine Busan Republic of Korea

**Keywords:** connectometry, diffusion tensor imaging, magnetic resonance imaging, obstructive sleep apnea, white matter

## Abstract

**Introduction:**

Using correlation tractography, this study aimed to find statistically significant correlations between white matter (WM) tracts in participants with obstructive sleep apnea (OSA) and OSA severity. We hypothesized that changes in certain WM tracts could be related to OSA severity.

**Methods:**

We enrolled 40 participants with OSA who underwent diffusion tensor imaging (DTI) using a 3.0 Tesla MRI scanner. Fractional anisotropy (FA), mean diffusivity (MD), axial diffusivity (AD), radial diffusivity (RD), and quantitative anisotropy (QA)‐values were used in the connectometry analysis. The apnea‐hypopnea index (AHI) is a representative measure of the severity of OSA. Diffusion MRI connectometry that was used to derive correlational tractography revealed changes in the values of FA, MD, AD, RD, and QA when correlated with the AHI. A false‐discovery rate threshold of 0.05 was used to select tracts to conduct multiple corrections.

**Results:**

Connectometry analysis revealed that the AHI in participants with OSA was negatively correlated with FA values in WM tracts that included the cingulum, corpus callosum, cerebellum, inferior longitudinal fasciculus, fornices, thalamic radiations, inferior fronto‐occipital fasciculus, superior and posterior corticostriatal tracts, medial lemnisci, and arcuate fasciculus. However, there were no statistically significant results in the WM tracts, in which FA values were positively correlated with the AHI. In addition, connectometry analysis did not reveal statistically significant results in WM tracts, in which MD, AD, RD, and QA values were positively or negatively correlated with the AHI.

**Conclusion:**

Several WM tract changes were correlated with OSA severity. However, WM changes in OSA likely involve tissue edema and not neuronal changes, such as axonal loss. Connectometry analyses are valuable tools for detecting WM changes in sleep disorders.

## Graphical abstract

Correlation of diffusion tensor tractography with obstructive sleep apnea severity Bong Soo Park, Dong Ah Lee, Ho‐Joon Lee, Jinseung Kim, Junghae Ko, Won Hee Lee, Jiyae Yi, Kang Min Park* Using correlation tractography, this study aimed to find statistically significant correlations between white matter (WM) tracts in participants with obstructive sleep apnea (OSA) and OSA severity. Connectometry analysis revealed that the AHI in participants with OSA was negatively correlated with FA‐values in WM tracts. Connectometry analyses are valuable tools for detecting WM changes in sleep disorders.

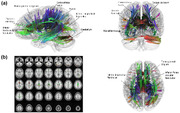

## 1 INTRODUCTION

Obstructive sleep apnea (OSA) is a condition characterized by the repeated collapse of the upper airway during sleep, leading to obstructive apnea, hypopnea, and potential respiratory effort‐related arousals (Gomase et al., 2023; Khor et al., 2023). OSA stands as the most prevalent sleep‐related breathing disorder, with estimated rates of 15%−30% in males and 10%−15% in females in North America (Bixler et al., 2001; Peppard et al., 2013). The prevalence of OSA appears to be on the rise, possibly attributed to increasing obesity rates and improved detection methods (Lechner et al., 2019; Lee et al., 2022; Peppard et al., 2013).

Patients with OSA typically exhibit normal results in observational brain analysis, but quantitative assessment through brain magnetic resonance imaging (MRI) reveals alterations in gray matter volume across various brain structures (Park & Kim, 2022; Shi et al., 2017). Additionally, studies have highlighted associations between OSA and changes in white matter (WM) tracts (Castronovo et al., 2014; Chen et al., 2015; Lee et al., 2019). OSA can compromise WM integrity in vulnerable regions, and this impairment correlates with increased disease severity. In a study involving 135 OSA patients, alterations in WM integrity and structural connectivity were observed in the middle cingulate and paracingulate gyri, the posterior cingulate gyrus, and amygdala. Global network properties and regional efficiency differed between OSA patients and healthy controls (Lee et al., 2019). Furthermore, using diffusion tensor imaging (DTI)‐MRI, patients with OSA displayed decreased fractional anisotropy (FA) values in the WM of specific brain regions, such as the right transverse temporal, anterior cingulate, and paracingulate gyri; left postcentral, middle frontal, and medial frontal gyri; as well as the putamen (Lee et al., 2019). Notably, no studies to date have specifically investigated statistically significant correlations between WM tracts and OSA severity.

The brain's connectome functions as a comprehensive map delineating cortical connections between different regions (Sporns, 2013; Turk‐Browne, 2013). The primary modality for assessing the structural connectome in humans is diffusion MRI, employing a fiber‐tracking algorithm to map macroscopic connections between gray matter parcellations (Akil et al., 2011; Seung, 2011; Sporns, 2013). Despite the growing popularity of diffusion MRI‐based tractography over the past decade, recent studies have raised concerns about the accuracy of measuring end‐to‐end connectivity. Fiber‐tracking algorithms, especially near gray matter targets, exhibit limited reliability, casting doubt on the effectiveness of “find‐difference‐in‐track” techniques (Reveley et al., 2015; Thomas et al., 2014). To overcome the limitations of end‐to‐end fiber tracking, a novel concept called the local connectome has emerged. The local connectome gauges connectivity between adjacent voxels within a white matter fascicle, utilizing spin density. Understanding the local orientation and integrity of fiber bundles within the core of white matter is crucial for discerning the origin and termination points of a bundle. Consequently, the local connectome functions as a fundamental unit of the end‐to‐end structural connectome and can serve as a surrogate for global connectivity analysis—an approach known as connectometry (Yeh et al., 2016). Connectometry adheres to a “track‐difference” paradigm, focusing on the segment of the fiber bundle demonstrating statistically significant associations with the variables under study, rather than mapping the entire end‐to‐end connectome. This involves reconstructing diffusion MRI data into a standard template space to create a local connectome matrix for a group of subjects. Subsequently, study variables are correlated with this matrix to identify connections with statistically significant correlations (Yeh, Badre et al., 2016). These localized connectomes are then tracked along the core pathway of the fiber bundle using a fiber‐tracking algorithm and compared to the null distribution of coherent associations through permutation statistics (Nichols & Holmes, 2002). A recent study employed connectometry to investigate neuronal injuries in individuals with mild traumatic brain injuries, showcasing the potential of this approach to illuminate the intricacies of brain connectivity and its association with various neurological conditions (Li et al., 2022).

A diffusion tensor offers various diffusivity measures, including FA, axial diffusivity (AD), radial diffusivity (RD), and mean diffusivity (MD) (Dong et al., 2020). AD reflects the rate of water diffusion parallel to axonal fibers, while RD indicates the rate of water diffusion perpendicular to axonal bundles. MD represents the average of the three eigenvalues of the tensor diffusivity. Well‐myelinated fibers typically exhibit high FA and low RD. However, demyelination leads to a substantial change in RD, and axonal loss results in decreased AD (Dong et al., 2020; Mohammadi et al., 2023). In contrast, generalized q‐sampling imaging (GQI) provides quantitative anisotropy (QA) and an isotropic diffusion component derived from GQI analysis (ISO), both based on diffusion density (Yeh et al., 2010). QA is calculated using peak orientations on a spin distribution function (SDF), where each orientation defines a specific QA value. Unlike FA, which is defined per voxel, QA is defined per fiber orientation. This distinction significantly impacts fiber tracking, with QA proving valuable in filtering out false fibers, particularly in scenarios involving crossing fibers (Guo et al., 2022; Yeh et al., 2010). It is essential to recognize that these two measures, one based on diffusivity and the other on density, hold distinct clinical meanings.

Utilizing correlation tractography, specifically focusing on FA and QA values, this study aimed to identify statistically significant correlations between white matter (WM) tracts in participants with obstructive sleep apnea (OSA) and the severity of OSA indicated by the apnea‐hypopnea index (AHI). The hypothesis posited that changes in specific WM tracts could be associated with the severity of OSA.

## 2 METHODS

### 2.1 Participants: patients with obstructive sleep apnea

This research took place at a tertiary care hospital and received approval from the Institutional Review Board. Forty participants meeting the OSA criteria outlined by Kapur et al. (2017) were included. These criteria encompassed the following: (1) a diagnosis of OSA confirmed by laboratory polysomnography (PSG) indicating an AHI > 5, coupled with symptoms such as sleepiness or chronic snoring, within the period from August 2018 to June 2023; (2) the absence of any other medical or neurological disorders, excluding OSA; (3) no observable structural brain lesions based on the observational analysis of brain MRI; and (4) possession of DTI‐MRI data obtained at the time of OSA diagnosis. Clinical and PSG data were gathered from participants with OSA, encompassing information such as age, sex, body mass index, Epworth sleepiness scale score, total sleep time, sleep efficiency, sleep stage ratios (N1, N2, N3, R), minimum oxygen saturation, total AHI during sleep, AHI during stage N, AHI during stage R, and the total respiratory disturbance index during sleep.

### 2.2 Diffusion tensor imaging‐MRI acquisition

Participants diagnosed with obstructive sleep apnea (OSA), who provided consent for the utilization of a 3.0 T MRI scanner for research purposes, underwent diffusion tensor imaging (DTI). The 3.0 T MRI scanner utilized a 32‐channel head coil (Achieva TX, Phillips Healthcare, Best, The Netherlands). The specific DTI parameters were as follows: 32 distinct diffusion directions, *b*‐values of 0 and 1000 s/mm^2^ (b0 images were acquired once), repetition time/echo time = 8620/85 ms, fractional anisotropy (FA) = 90°, slice thickness = 2.25 mm, acquisition matrix = 120 × 120, field of view = 240 × 240 mm^2^, and parallel imaging with sensitivity encoding (SENSE) at a factor of 2. The phase direction was set in the anterior‐posterior direction, and the fat shift occurred in the posterior direction.

### 2.3 Statistical analysis of the connectometry analysis

**FIGURE 1:**
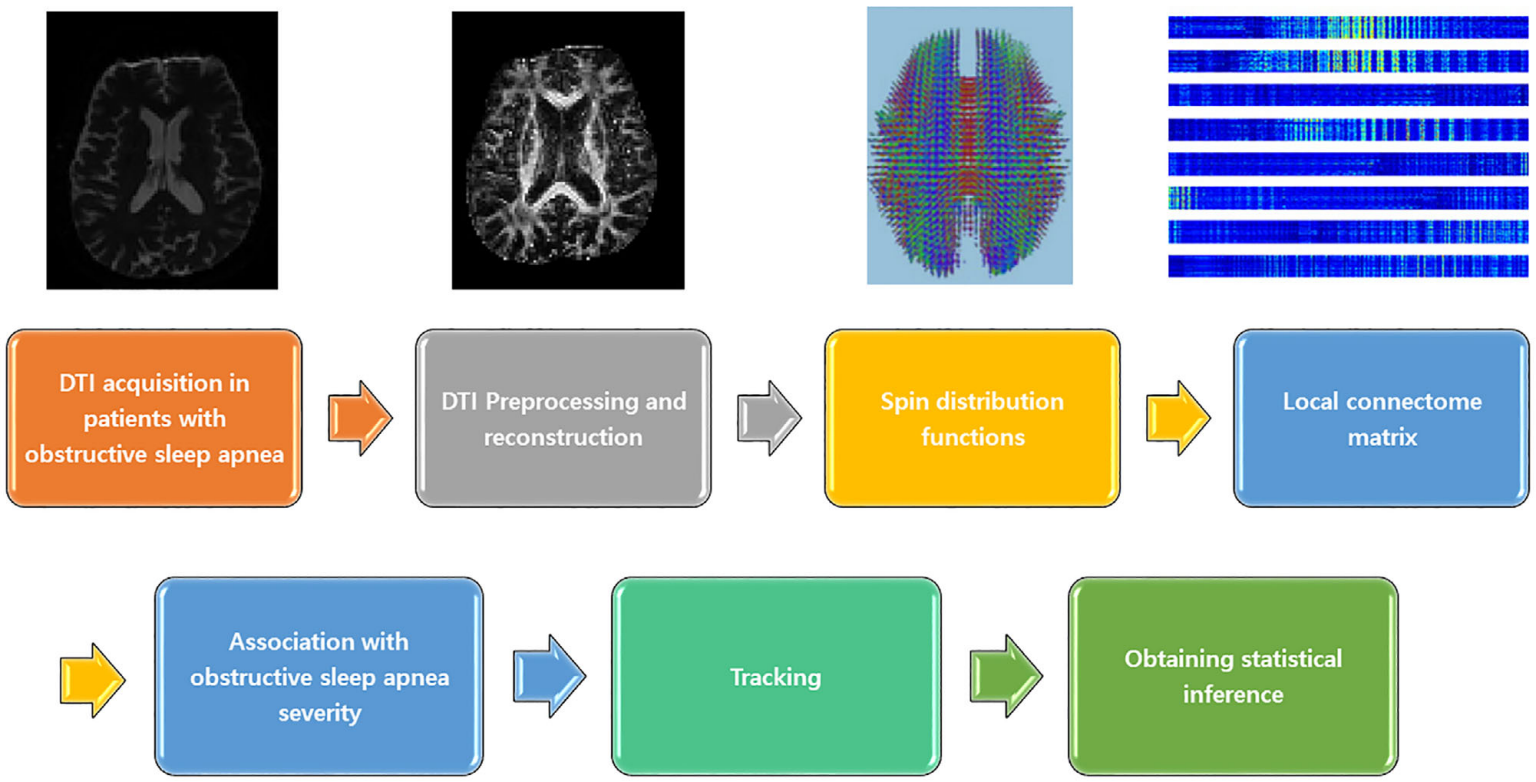
The process for the connectometry analysis in this study is depicted. DTI, diffusion tensor imaging.

Figure 1 illustrates the connectometry analysis process employed in this study. The Connectometry Database incorporated 40 diffusion MRI scans from participants diagnosed with obstructive sleep apnea (OSA). For DTI preprocessing of brain MRI, the DSI Studio software (version May 2022, http://dsi‐studio.labsolver.org) was utilized. This program, equipped with open‐source images, addressed eddy current and phase distortion artifacts, generated a mask through thresholding, smoothing, and defragmentation, and underwent a quality control step for DTI. Subsequently, diffusion data were reconstructed using the DTI method and generalized q‐sampling imaging (GQI) with a diffusion sampling length ratio of 1.25 (Yeh et al., 2010). The connectometry analysis incorporated DTI‐based metrics, including FA, MD, AD, and RD values, and GQI‐based QA values, extracted as the local connectome fingerprint (Yeh, Vettel et al., 2016). When correlated with the apnea‐hypopnea index (AHI), a representative measure of OSA severity, diffusion MRI connectometry demonstrated changes in FA, MD, AD, RD, and QA values (Table 1). Non‐parametric Spearman partial correlation, removing the effects of sex and age via a multiple regression model, was used to derive these correlations. A T‐score threshold of 2.5 guided deterministic fiber tracking for correlational tractography (Yeh, Vettel et al., 2016). In the post connectometry analyses, partial correlation analysis adjusted with age and sex was performed by calculating the average of the FA values of the tracts that significantly correlated with the AHI, and expressed in a graph.

**TABLE 1:** Diffusivity parameters of diffusion tensor imaging in this study.

	Parameters	Model	Interpretation
FA	Fractional anisotropy	Diffusion tensor	Associated with axonal integrity
AD	Axial diffusivity	Diffusion tensor	Associated with axonal density
RD	Radial diffusivity	Diffusion tensor	Associated with myelination
MD	Mean diffusivity	Diffusion tensor	Associated with edema and cell infiltration
QA	Quantitative anisotropy	Q‐space imaging	Associated with axonal density

Parameters were set for the entire brain region without excluding specific regions from the analysis. The tracts were filtered through topology‐informed pruning with four iterations (Yeh et al., 2019). These local connectomes were then tracked along the core pathway of a fiber bundle using a fiber‐tracking algorithm and compared with a null distribution of coherent associations through permutation statistics. A false‐discovery rate threshold of 0.05 was applied to select tracts for multiple corrections, with 4000 randomized permutations used for the group labels to obtain a null distribution of track length. In addition, the sample size for correlation analysis was analyzed using alpha of 0.05, beta of 0.20, and a correlation coefficient of 0.568, indicating a minimum required sample size of 22 (MedCalc^®^ Statistical Software version 22.013, MedCalc Software Ltd, Ostend, Belgium; https://www.medcalc.org; 2023).

## 3 RESULTS

### 3.1 Participants

Table 2 shows the clinical and PSG findings of participants with OSA included in this study. Of the 40 participants with OSA, 27 (67.5%) were male, and 13 (32.5%) were female. Eighteen participants (45.0%) had mild OSA (5 ≤ AHI ≤ 15), 13 (32.5%) had moderate OSA (15 < AHI ≤ 30), and 9 (22.5%) had severe OSA (AHI > 30).

**TABLE 2:** Clinical and polysomnographic data in participants with obstructive sleep apnea.

	Participants with obstructive sleep apnea (*N* = 40)
Clinical data	
Mean age ± SD, years	61.5 ± 14.8
Male/Female, *N* (%)	27 (67.5)/13 (32.5)
Median Body mass index (interquartile range), kg/m^2^	25.2 (23.9–27.1)
Median Epworth Sleepiness Scale (interquartile range)	6 (3−9)
Polysomnographic data	
Mean Total Sleep Time ± SD, minutes	349.5 ± 54.3
Median Sleep Efficiency (interquartile range), %	77.1 (71.8−85.1)
Median Stage N1 (interquartile range), %	25.3 (13.0−31.2)
Median Stage N2 (interquartile range), %	54.1 (46.9−64.4)
Median Stage N3 (interquartile range), %	2.0 (0.4−9.5)
Median Stage R (interquartile range), %	15.3 (7.9−21.6)
Median minimum oxygen saturation (interquartile range), %	85.0 (82.5–88.7)
Median Total AHI (interquartile range)	15.4 (9.9−24.9)
Median AHI during stage N (interquartile range)	15.8 (8.7−25.4)
Median AHI during stage R (interquartile range)	13.5 (6.4−27.1)
Median Total RDI (interquartile range)	18.7 (12.7−28.7)

SD, standard deviation; AHI, apnea‐hypopnea index; RDI, respiratory disturbance index.

### 3.2 Correlation of diffusion tensor tractography with OSA severity

**FIGURE 2:**
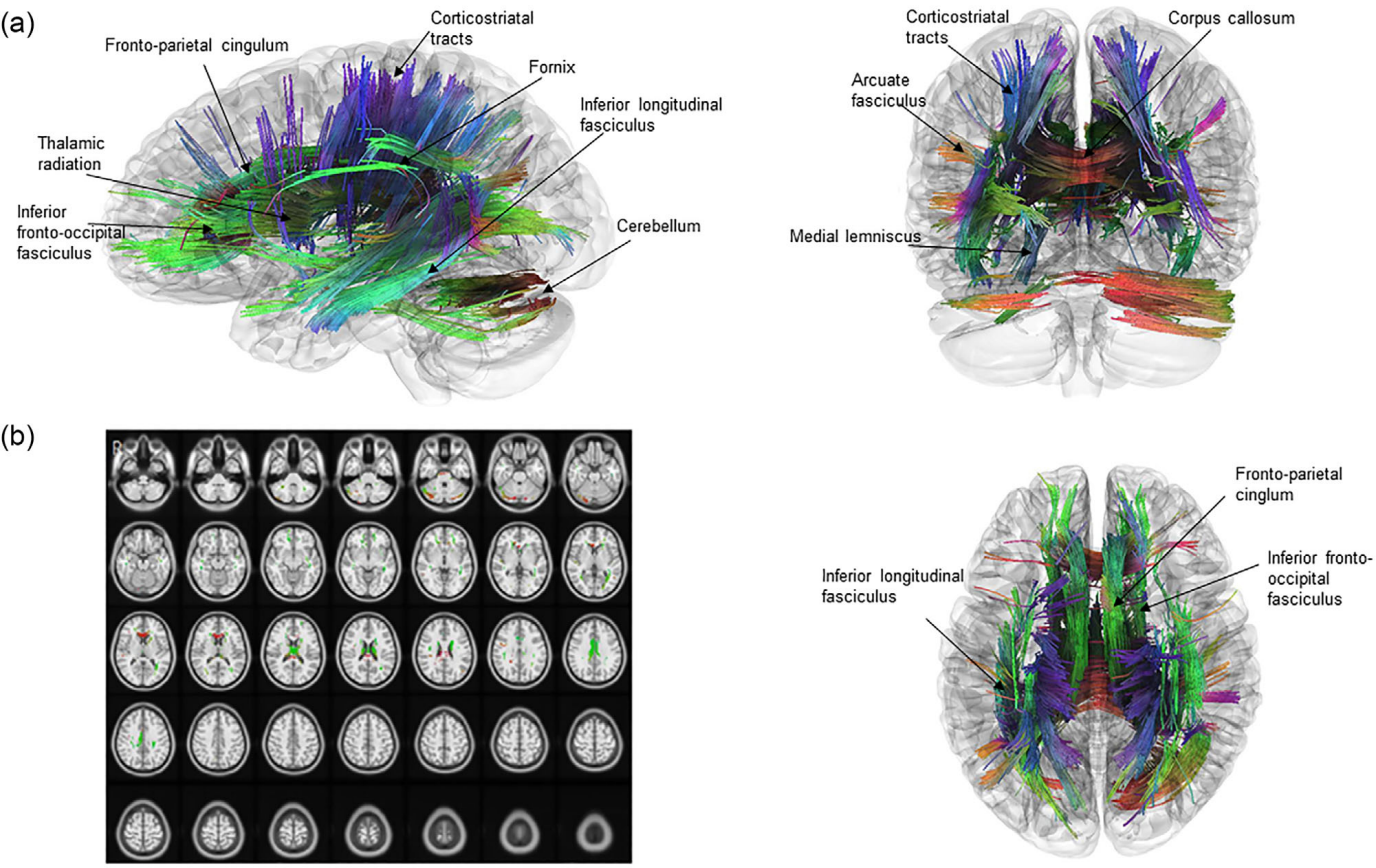
Tracts with fractional anisotropy (FA) negatively correlated with apnea‐hypopnea index (AHI) are shown. A false‐discovery rate threshold of 0.05 has been used. Connectometry analysis reveals that the AHI in participants with OSA is negatively correlated with FA values in the following WM tracts: the bilateral fronto‐parietal cingulum; the body, tapetum, and forceps minor of the corpus callosum; right cerebellum; left inferior longitudinal fasciculus; bilateral superior and posterior thalamic radiations; left anterior thalamic radiation; right cingulum parolfactory; left inferior fronto‐occipital fasciculus; bilateral fornices; left posterior and superior corticostriatal tracts; right parahippocampal cingulum; bilateral medial lemnisci; and left arcuate fasciculus (a). Tracts that show a strong correlation with OSA severity have been projected onto T1‐weighted images (b).

Connectometry analysis did not reveal statistically significant results in WM tracts, in which FA values were positively correlated with the AHI. However, connectometry analysis revealed that the AHI in participants with OSA was negatively correlated with FA values in the following WM tracts: the bilateral fronto‐parietal cingulum; the body, tapetum, and forceps minor of the corpus callosum; right cerebellum; left inferior longitudinal fasciculus; bilateral superior and posterior thalamic radiations; left anterior thalamic radiation; right cingulum parolfactory; left inferior fronto‐occipital fasciculus; bilateral fornices; left posterior and superior corticostriatal tracts, right parahippocampal cingulum; bilateral medial lemnisci; and left arcuate fasciculus (Figure 2).

Connectometry analysis did not reveal statistically significant results in WM tracts, in which MD, AD, RD, and QA values were not positively or negatively correlated with the AHI.

### 3.3 Post connectometry analyses

**FIGURE 3:**
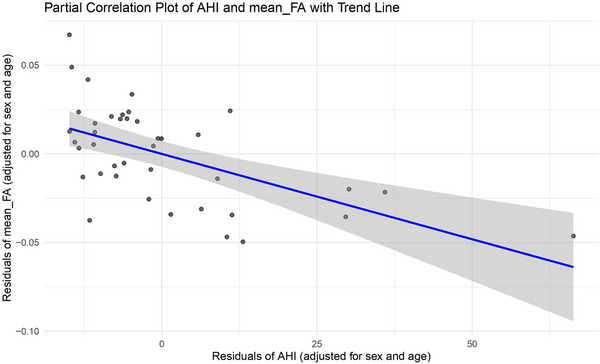
Correlation plot between mean fractional anisotropy (FA) of the statistically significant correlated WM tracts and apnea‐hypopnea index (AHI) in participants with obstructive sleep apnea is depicted. The figure shows a statistically significant negative correlation between the mean FA and the AHI (*r* = −0.568, *p* < .001).

Figure 3 shows the correlation plot between mean FA of the significantly correlated white matter tracts and AHI in patients with OSA (*r* = −0.568, *p *< .001).

## 4 DISCUSSION

In this investigation, we identified specific WM tracts in participants with OSA that exhibited associations with OSA severity. The AHI in individuals with OSA demonstrated a negative correlation with FA in WM tracts encompassing the cingulum, corpus callosum, cerebellum, inferior longitudinal fasciculus, fornices, thalamic radiations, inferior fronto‐occipital fasciculus, superior and posterior corticostriatal tracts, medial lemnisci, and arcuate fasciculus. However, no statistically significant results were observed in WM tracts where the AHI was positively correlated with FA values. Furthermore, the connectometry analysis did not unveil statistically significant outcomes in WM tracts where MD, AD, RD, and QA values were positively or negatively correlated with the AHI.

Our findings align with previous research that has reported a negative correlation between the AHI and FA values in various brain areas, including the medial temporal, parietal, superior longitudinal fasciculus, corticospinal tract, and fronto‐occipital fasciculus (Chen et al., 2020, 2015). FA, a measure reflecting the degree of directionality in water movement, indicates white matter damage, with lower FA values associated with such damage (Baril et al., 2021). Despite reports of decreased FA in OSA patients regardless of severity (Koo et al., 2020; Lee et al., 2019), other diffusivity‐related measures exhibit varying results contingent upon pathological stages and patient characteristics (Baril et al., 2021). Our study is the pioneering exploration of the relationship between AHI and QA values in individuals with OSA. No statistically significant results were observed for WM tracts where QA values were positively or negatively correlated with AHI. It is important to note the distinction between FA and QA. While the myelination of axons inhibits diffusion, the DTI model cannot effectively account for this effect. DTI metrics, including FA, AD, and MD, are influenced by various biological changes, making them prone to substantial variation, often necessitating a large sample size for statistical significance (Yeh et al., 2013). QA, however, relies on q‐space imaging to ascertain the densities of restricted and less‐restricted diffusion, offering distinct advantages. The GQI length parameter specifies the distance scale for evaluating restricted diffusion, allowing for a clear separation between restricted and less‐restricted diffusion even in low signal‐to‐noise ratio conditions. QA's ability to measure the density of anisotropic diffusing water makes it more resistant to inflammation and edema, as demonstrated in a neurosurgical study highlighting its resistance to peritumoral edema (Zhang et al., 2013). Furthermore, QA quantifies anisotropy for each fiber population individually, providing a measurement for each fiber, unlike FA, which is shared by all fiber populations within a voxel. QA values are less affected by crossing fibers and partial volume effects, exhibiting better resolution and increased sensitivity to physiological variations (M. Li et al., 2022, 2021). In clinical applications, diffusivity measurements like FA values demonstrate higher sensitivity to pathological conditions, whereas density measurements such as QA values exhibit heightened sensitivity to physiological variations (Yeh, Vettel et al., 2016). A phantom study corroborated QA's resistance to free water effects and partial volume of crossing fibers compared to FA (Yeh et al., 2013). Clinical case studies further validated QA‐based tractography as more resistant to peritumoral edema, offering greater reproducibility and uniqueness compared to diffusivity‐based measurements (Zhang et al., 2013).

FA values exhibit a decrease during acute neuronal injury and chronic neurodegeneration. While FA is highly sensitive, its specificity is low, as a decline in FA values may stem from various factors, including vasogenic edema, demyelination, inflammation, and axonal degeneration. Frequently used as an initial screening method, FA's sensitivity makes it prone to detecting changes beyond axonal loss. In contrast, QA values are more specific to axonal loss, with their stability in response to acute axonal injury or edema. In cases of acute neuronal injury or inflammation, a concurrent decrease in FA values and stability in QA values may suggest reversible axonal injury. Understanding the differences between FA and QA, regions in the brain with decreased FA but unchanged QA may indicate tissue edema without axonal injury, while regions with unchanged FA but decreased QA could signify alterations in diffusion patterns due to transient permeability changes in myelin, possibly linked to neuroplasticity (Guo et al., 2022; Jin et al., 2019; Yeh et al., 2010; Zhang et al., 2013). In the current study, brain regions displaying a statistically significant correlation with FA showed no corresponding relation to QA, suggesting that observed changes in WM in participants with OSA may be attributed to brain edema. These findings align with a prior study linking impairments in cognitive domains, mood, and sleepiness to a diffuse reduction in WM fiber integrity, reflected by decreased FA and MD values in various brain regions of pretreatment patients with OSA. Interestingly, after 3 months of continuous positive airway pressure (CPAP) treatment, only minor WM changes were observed. However, over a 12‐month CPAP treatment period, patients with OSA demonstrated nearly complete reversal of WM abnormalities in all affected regions. These results suggest that WM changes in OSA participants may involve tissue edema rather than structural injury to the WM tract, and they can be effectively and reversibly addressed by OSA treatments such as CPAP. Consequently, our study underscores the importance of active treatment, such as CPAP, for individuals with OSA.

This study had some limitations. First, because this study had a cross‐sectional design, changes in the values of FA or QA, after the administration of OSA treatments, such as CPAP, could not be studied. Considering that QA reveals a high degree of individuality, the intersubject variance of QA can be quite high (Yeh, Vettel et al., 2016). Thus, although connectometry analysis can also be beneficial in cross‐sectional studies, it is best suited for longitudinal studies. Second, as the sample size was small and the study was conducted in one tertiary hospital, it may be difficult to generalize the results. Nevertheless, this is the first study that investigated the relationship between correlational tractography using QA and FA values and OSA severity, and proved that correlation tractography could be useful in the future research of neurological diseases, including sleep disorders.

## 5 CONCLUSION

Connectometry analysis does not reveal significant results in WM tracts, in which FA values are not positively correlated with the AHI, but it reveals that the AHI in participants with OSA is negatively correlated with FA values in the several WM tracts. In addition, connectometry analysis does not reveal significant results in WM tracts, in which MD, AD, RD, and QA values are not positively or negatively correlated with the AHI. It suggests that WM changes in OSA likely involve tissue edema and not neuronal changes, such as axonal loss. Connectometry analyses, such as correlation tractography, are valuable tools in the detection of WM changes in sleep disorders.

## AUTHOR CONTRIBUTIONS

BS Park, DA Lee, and KM Park: conception and design. HJ Lee, J Kim, J Ko, WH Lee, and J Yi: acquisition of data, analysis, and interpretation of data. BS Park, DA Lee, and KM Park: drafting the manuscript or revising. KM Park: final approval

## CONFLICT OF INTEREST STATEMENT

All authors have no conflicts of interest to declare at the time of submission.

## FUNDING

This study received support from the National Research Foundation of Korea (NRF) grant funded by the Korean government (MSIT) (Grant No. NRF‐2022R1F1A1074160).

### PEER REVIEW

The peer review history for this article is available at https://publons.com/publon/10.1002/brb3.3541.

## Data Availability

The data that support the findings of this study are available from the corresponding author upon reasonable request.
